# Regulatory pathways and drugs associated with ferroptosis in tumors

**DOI:** 10.1038/s41419-022-04927-1

**Published:** 2022-06-10

**Authors:** Dan Wang, Le Tang, Yijie Zhang, Guili Ge, Xianjie Jiang, Yongzhen Mo, Pan Wu, Xiangying Deng, Lvyuan Li, Sicheng Zuo, Qijia Yan, Shanshan Zhang, Fuyan Wang, Lei Shi, Xiayu Li, Bo Xiang, Ming Zhou, Qianjin Liao, Can Guo, Zhaoyang Zeng, Wei Xiong, Zhaojian Gong

**Affiliations:** 1 NHC Key Laboratory of Carcinogenesis and Hunan Key Laboratory of Cancer Metabolism, Hunan Cancer Hospital and the Affiliated Cancer Hospital of Xiangya School of Medicine, Central South University, Changsha, China; 2 Key Laboratory of Carcinogenesis and Cancer Invasion of the Chinese Ministry of Education, Cancer Research Institute, Central South University, Changsha, China; 3 Department of Stomatology, Xiangya Hospital, Central South University, Changsha, China; 4 Department of Oral and Maxillofacial Surgery, the Second Xiangya Hospital, Central South University, Changsha, China; 5 Hunan Key Laboratory of Nonresolving Inflammation and Cancer, Disease Genome Research Center, The Third Xiangya Hospital, Central South University, Changsha, China

**Keywords:** Cancer, Cell death, Non-coding RNAs

## Abstract

Ferroptosis is a type of cell death that depends on iron and reactive oxygen species (ROS). The accumulation of iron and lipid peroxidation primarily initiates oxidative membrane damage during ferroptosis. The core molecular mechanism of ferroptosis includes the regulation of oxidation and the balance between damage and antioxidant defense. Tumor cells usually contain a large amount of H_2_O_2_, and ferrous/iron ions will react with excessive H_2_O_2_ in cells to produce hydroxyl radicals and induce ferroptosis in tumor cells. Here, we reviewed the latest studies on the regulation of ferroptosis in tumor cells and introduced the tumor-related signaling pathways of ferroptosis. We paid particular attention to the role of noncoding RNA, nanomaterials, the role of drugs, and targeted treatment using ferroptosis drugs for mediating the ferroptosis process in tumor cells. Finally, we discussed the currently unresolved problems and future research directions for ferroptosis in tumor cells and the prospects of this emerging field. Therefore, we have attempted to provide a reference for further understanding of the pathogenesis of ferroptosis and proposed new targets for cancer treatment.

## Facts

- Ferroptosis regulates the balance between oxidative damage and antioxidant defense.
- It is a type of programmed cell death dependent on iron-mediated oxidative damage.
- Some ncRNAs related to ferroptosis are mainly miRNA, lncRNAs, and circRNAs.

## Open questions

- What is the crosstalk between ferroptosis and other cell death pathways?
- To what extent does lipid peroxidation induce ferroptosis?
- Are ferroptosis inducers effective in killing tumor cells in clinical therapy?
- What is the progress of ferroptosis induced by nanomaterials in tumor therapy?

## Introduction

Cell death has long been recognized as a characteristic of malignancy [1, 2]. As early as the 1960s, the concept of programmed cell death (PCD) was introduced. Cell death is regulated by molecular mechanisms to ensure homeostasis and normal development of the body. Imbalance in this regulation leads to the appearance of various pathological symptoms in the body [3, 4]. In the following decades, new forms of cell death, such as apoptosis, necroptosis, pyroptosis, and autophagy were discovered, each with its unique mechanism [5, 6]. Apoptosis refers to cells in certain physiological or pathological conditions, controlled by internal genetic mechanism, in accordance with their own procedures of initiative, physiological death process. It is regulated by apoptosis-related genes, such as Bcl-2, P53, cytochrome C (Cyt C), APAF-1, and the caspase family proteins [7]; Necroptosis is a regulated form of death mediated by RIP1 and RIP3 kinases. Features include early loss of plasma membrane integrity, leakage of cell contents, and swelling of organelles. necroptosis, neither necrosis nor apoptosis, is an alternative modulated cell death model that simulates the characteristics of apoptosis and necrosis [8]. Pyroptosis is programmed cell necrosis mediated by Gasdermin D. It mainly relies on inflammasome to activate the part of the proteins in the caspase family, causing them to cut Gasdermin D protein, activate Gasdermin D protein, and the activated Gasdermin D protein is translocated to the membrane to form holes. The cell expands until its membrane ruptures, causing the release of its contents and triggering an intense inflammatory response [9, 10]. Autophagy plays a key role in cell and tissue homeostasis. Many stimuli, such as nutrient deficiency, oxidative stress, and protein aggregation, can kick-start autophagy. It can be divided into three subtypes: macro-autophagy, microautophagy, and chaperone-mediated autophagy [11].

In 2012, scientists named a new mode of regulated cell death (RCD) induced by the ferrous ion (Fe^2+^)-dependent accumulation of lipid peroxides as ferroptosis [12–17]. The morphological and biochemical characteristics of ferroptosis are distinct from other types of PCDs (e.g., apoptosis, necroptosis, Pyroptosis, and autophagy) [18]. In terms of biochemical characteristics, cells undergoing ferroptosis show an imbalance of oxidation–reduction levels: a significant increase in intracellular reactive oxygen species (ROS) and a significant decrease in NADPH, and therefore lipid antioxidants can inhibit ferroptosis. Cancer cells accumulate high levels of iron and ROS to promote their metabolic activities and growth [19]. Mitochondria are important sites of ROS generation and fatty acid metabolism and provide specific lipid precursors for ferroptosis to occur in the cell. Therefore, mitochondria as iron-rich (iron is necessary for the respiratory chain), ROS generating organelles, are considered as significant locations for ferroptosis. Morphologically, mitochondria undergoing ferroptosis show distinct changes compared to those in normal cells, which can be observed using transmission electron microscopy. It can be clearly seen that the volume of mitochondria in the cell reduces, the boundary shrinks, and the cristae reduce or disappear. The shape of mitochondria changes from a long rod shape to a punctate shape along with a ruptured outer membrane [20–22]. Further, during ferroptosis, no apoptotic bodies appear, and characteristic apoptotic proteins such as Caspases, Bax, and Bak are not activated; hence, apoptosis inhibitors cannot prevent ferroptosis [14, 23]. Ferroptosis can be inhibited to a certain extent by necroptosis inhibitors [24]. However, ferroptosis could not be inhibited by inhibitors of apoptosis, pyroptosis, and autophagy, but by iron-chelating agents and antioxidants [25]. Studies have found that different forms of RCD have different molecular mechanisms and modes of death, and there are interactions and influences among various forms of RCD [26]. Ferroptosis involves a series of complex biochemical reactions, gene expression, and signal transduction. It has gained widespread attention and is expected to bring a breakthrough in the treatment of several diseases, including cancer [27–30].

## The antioxidant systems and execution systems of ferroptosis

**Fig. 1:**
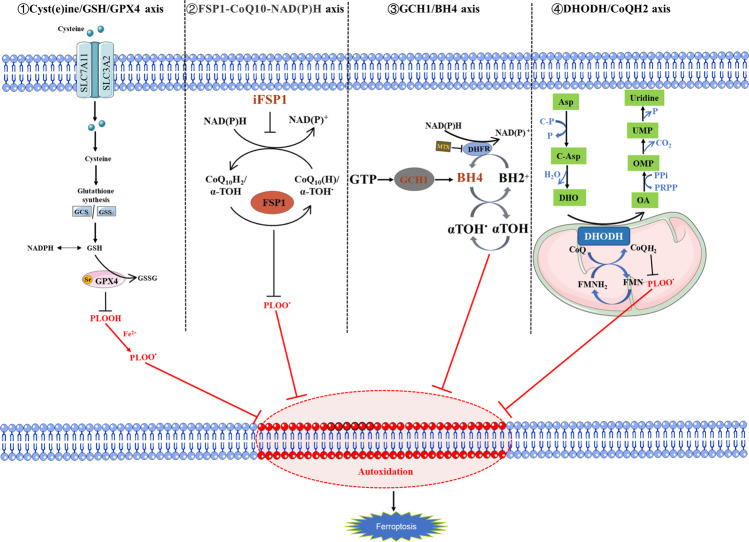
Four ferroptosis defense pathways. Four ferroptosis defense pathways have been found so far, namely, Cyst(e)ine/GSH/GPX4 axis, FSP1-CoQ10-NAD(P)H axis, GCH1/BH4 axis, and DHODH/CoQH2 axis; ① GPX4 specifically catalyzes the loss of oxidative activity of lipid peroxides through glutathione (GSH)-dependent manner, and protects cells from the threat of ferroptosis is the most classic way; ② as a phospholipid peroxidation inhibitor that does not rely on glutathione, FSP1 converts ubiquinone on the cell membrane into reduced ubiquinol, which can inhibit peroxidation and prevent ferroptosis; ③ the GCH1/BH4 pathway acts as an endogenous antioxidant pathway, GCH1 protects cells from ferroptosis primarily through the antioxidant action of BH4; ④ DHODH resists ferroptosis in mitochondria by regulating the production of dihydroubiquinone in the inner membrane of mitochondria.

The regulation of ferroptosis is mainly competition between ferroptosis antioxidant defense system and ferroptosis execution system. Ferroptosis antioxidant defense system mainly divided into GPX4-dependent systems (SLC7A11/GSH/GPX4 axis) and GPX4-non-dependent systems (FSP1-CoQ10-NAD(P)H axis, GCH1/BH4 axis, and DHODH/CoQH2 axis) [31, 32] (Fig. 1).

### SLC7A11/GSH/GPX4 axis

The solute carrier family 7-member 11-glutathione-GPX4(SLC7A11-GSH-GPX4) signaling axis is one of the most classic ferroptosis defense pathways [33–35]. The l-cystine/glutamate antiporter SLC7A11 introduces cystine from the extracellular environment, which is transferred to the cytoplasm and converted into cysteine through a reduction reaction that consumes NADPH. Cysteine is subsequently used for glutathione (GSH) biosynthesis (and other biomolecules) and is involved in antioxidant defense mechanism, it is considered to be the rate-limiting precursor of the antioxidant glutathione [36] . GSH is a tripeptide composed of three amino acids (cysteine, glutamate, and glycine), which exists in a reduced (GSH) or an oxidized state (GSSG). These two states are important metabolites that maintain intracellular redox homeostasis and inhibit cell ferroptosis [37, 38]. GPX4 is the core regulatory protein of ferroptosis and has a unique role in inhibiting lipid peroxidation [24, 33]. It can reduce complex lipid peroxides to alcohols or convert free hydrogen peroxide into the water to prevent ferroptosis [33, 39, 40]. It can catalyze the oxidation of GSH to GSSG and peroxidize reduced polyunsaturated fatty acids, thereby protecting the phospholipid bilayer in the cell from oxidative damage and inhibiting ferroptosis. When the GSH-dependent lipid peroxide repair system is damaged, it causes ferroptosis through the fatal accumulation of lipid-reactive oxygen species [20, 41–43].

### GCH1/BH4 axis

GTP cyclohydrolyse-1 (GCH1) is the rate-limiting enzyme of the 6(R)-l-erythro-5,6,7,8-tetrahydrobiopterin (BH4) complex. Kraft found that the expression of GCH1 triggered the production of potent antioxidant BH4, thus preventing lipid peroxidation [44]; BH4 has powerful antioxidant capacity and can act as a direct antioxidant to protect cells from lipid peroxidation, it can also be used for de novo synthesis of CoQ10, which also protects cells from ferroptosis [45, 46]. The GCH1/BH4 pathway acts as an endogenous antioxidant pathway, GCH1 protects cells from ferroptosis primarily through the antioxidant action of BH4, and is completely independent of GPX4-mediated protection against ferroptosis [44]. GCH1 determines the level of BH4, highlighting a direct correlation between the GCH1/BH4 axis and inhibition of ferroptosis [47–49].

### FSP1-CoQ10-NAD(P)H axis

The pharmacological targeting of FSP1 has a strong synergistic effect with GPX4 inhibitors, which can trigger the ferroptosis of multiple tumor entities [24, 45]. There is a classic myristoylated motif at the N-terminus of ferroptosis suppressor protein 1 (FSP1), and the existence of this motif indicates that FSP1 may be related to lipid biomolecules. The mutation of the myristoylation site in FSP1 induced ferroptosis proving that FSP1 can only resist ferroptosis in the myristoylated form [46]. Ubiquinone, also known as coenzyme Q (CoQ10), exists in lipid membranes. It helps ATP production in mitochondria, and its reduced form is called ubiquinol. FSP1 converts the ubiquinone on the cell membrane into its reduced form ubiquinol, which can inhibit peroxidation and prevent ferroptosis [50]. NAD(P)H, one of the principal reductants, is produced by the pentose phosphate pathway (PPP) and can be phosphorylated by NAD kinase (NADK) to synthesize NADPH, which inhibits peroxidation damage caused by ferroptosis. The FSP1-CoQ10-NAD(P)H pathway exists as an independent parallel system, compatible with GPX4, and works with glutathione to inhibit phospholipid peroxidation and ferroptosis.

### DHODH/CoQH2 axis

Dihydroorotate dehydrogenase (DHODH) is a flavin-dependent enzyme located in the inner membrane of mitochondria. Its main function is to catalyze the fourth step of the pyrimidine biosynthesis pathway. That is, dihydroorotate (DHO) is oxidized to orotate (OA), and at the same time electrons are transferred to ubiquinone in the inner membrane of mitochondria, which is reduced to dihydroubiquinone [51]. Mao et al. reported that DHODH (Dihydroorotate dehydrogenase) coordinates with GPX4 by reducing ubiquitin formation in cancer cells, blocking the action of ferroptosis in mitochondrial intima [52], DHODH inhibitors induce ferroptosis and significantly inhibit tumor growth in solid tumors with low GPX4 expression, and the combination of ferroptosis inducer sulfasalazine and DHODH inhibitors has a good therapeutic effect in solid tumors with high GPX4 expression. DHODH/CoQH2 axis is an ferroptosis inhibitor independent of the classical GPX4 signaling pathway and based on mitochondrial lipid peroxidation of ferroptosis. The findings provide a new strategy for targeting ferroptosis in cancer treatment [52, 53].

## The lipid peroxidation of ferroptosis

ACSL protein mainly exists on the endoplasmic reticulum and the outer mitochondrial membrane and is a fatty acid activating enzyme. It is mainly responsible for the conversion of long-chain fatty acids to their active form acyl-CoA for their oxidation and lipid biosynthesis. Specifically, ACSL4 has a preference for long-chain polyunsaturated fatty acids, such as arachidonic acid (AA) or adrenergic acid (Ada) [54, 55], and converts them to arachidonic CoA and adrenal CoA, respectively, which are more likely to be oxidized to form lipid peroxides [56, 57].

Under normal circumstances, the accumulation of AA in cells is much lower than that of other fatty acids. The expression of ACSL4 upregulation is considered a biomarker and contributor to ferroptosis, it can esterify free polyunsaturated fatty acids into membrane phospholipids with the help of lysophosphatidylcholine acyltransferase 3 (LPCAT3). Synthesis of PUFA-PL mediated by LPCAT3 and ACSL4, as well as ALOX- and POR-mediated PUFA-PL peroxidation are necessary for ferroptosis to occur. Exogenous supplementation of AA/Ada (and other long-chain polyunsaturated fatty acids) can make ACSL4 knockout cells sensitive to ferroptosis. Hydroxyl radicals can catalyze the peroxidation of various biological macromolecules in cells, including polyunsaturated fatty acids (PUFA), and it is known that various phospholipid bilayers perform important biological functions in cells. The basic unit of phospholipid is composed of hydrophilic phosphoglycerol and hydrophobic polyunsaturated fatty acid chains. The peroxidation of the PUFA chains leads to the destruction of the phospholipid bilayer membrane structure of the cell enhancing membrane permeability, which ultimately leads to cell death [58, 59].

## Iron metabolism

**Fig. 2:**
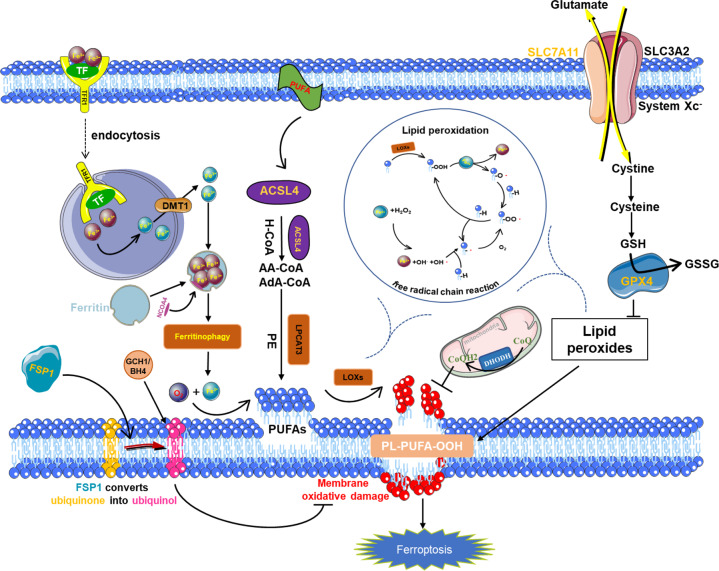
Core molecular machinery and regulatory pathways of ferroptosis. System X_C_- obtains cystine from the extracellular environment. The cystine introduced into the cells is converted into cysteine in the cytoplasm through a reduction reaction that consumes NADPH; cysteine is then used for the biosynthesis of glutathione, Glutathione peroxidase 4 (GPX4) can catalyze reduced glutathione (GSH) to oxidized form (GSSG), at the same time, the peroxidized polyunsaturated fatty acids are reduced to protect the phospholipid bilayer in the cell from oxidative damage and inhibit ferroptosis; while the long-chain acyl-CoA synthase 4 (ACSL4) can reduce arachidonic acid and adrenal acid. Polyunsaturated fatty acids (PUFA) are activated to arachidonic CoA and adrenal CoA, which are more likely oxidized to form lipid peroxides and induce ferroptosis; membrane transferrin receptor 1 (transferrin receptor 1, TFR1) can transport trivalent iron ions into the cell, and under the action of divalent metal transporter1 (DMT1), it can convert Fe^3+^ to Fe^2+^, and excess iron is stored in ferritin. Nuclear receptor coativator 4 (NCOA4) mediates the degradation of ferritin in autophagosomes, resulting in the release of ferritin-bound iron as free Fe^2+^ (ferritinophagy), promoting ferroptosis; FSP1 converts ubiquinone to ubiquinol, alleviating persistent oxidative damage to cell membranes and inhibiting ferroptosis.

Compared with non-malignant cells, the growth of cancer cells is strongly dependent on the micronutrient iron (ferrum in Latin), which is necessary for the ferroptosis process and can be inhibited by various iron-chelating agents. The excessive iron load may lead to ferroptosis in cancer patients [60–62], membrane transferrin receptor 1 (TFR1) can transport Fe^3+^ ions into cells [63], was also recently identified as a biomarker for ferroptosis [63]. Under the action of DMT1, Fe^3+^ is converted to Fe^2+^. Excess iron is stored in ferritin, which includes a ferritin light chain (FTL) and ferritin heavy chain 1 (FTH1) [64, 65]. Therefore, ferritin abundance, especially ferritin heavy chain (FTH1) abundance, is critical for inhibiting ferroptosis [66]. Intracellular unstable iron (Fe2+) can produce a large number of ROS through the Fenton reaction, providing sufficient raw materials for lipid peroxidation, and it is also a cofactor of lipid peroxidase (ALOXs and POR), which determines the activity of these enzymes (Fig. 2).

## Tumor-related signaling pathways in ferroptosis

### NRF2

The nuclear factor-erythroid factor 2-related factor 2 (NRF2) signaling pathway is an important defense mechanism against ferroptosis, which needs to be activated to exert its antioxidant properties. NRF2 activity is affected by some related regulatory factors during ferroptosis. Subsequently, the activated NRF2 induces and regulates the expression of a series of downstream antioxidant factors. Therefore, in the NRF2 antioxidant regulatory factors category, both activation-related regulatory factors and downstream antioxidant factors or antioxidant systems are included.

**Fig. 3:**
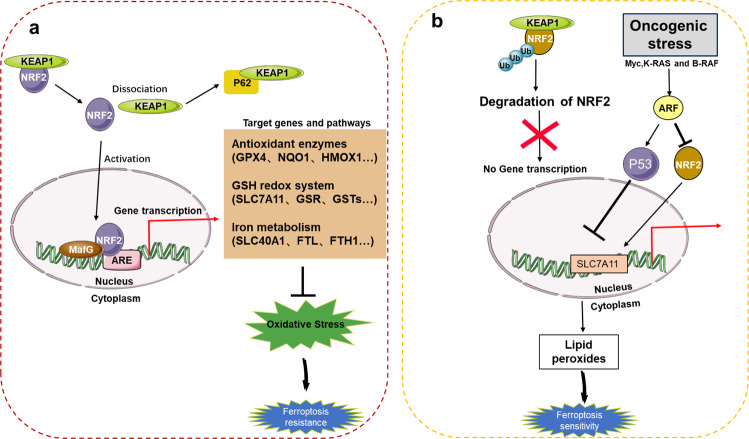
NRF2 and the ferroptosis network. **a** p62-mediated degradation of KEAP1 contributes to the activation of NRF2 in ferroptosis. The activated NRF2 enters the nucleus to initiate the transcription of antioxidant enzymes, glutathione redox system, iron metabolism, and other related molecules, alleviating oxidative stress, making tumor cells resistant to ferroptosis. **b** When NRF2 is degraded by ubiquitination, it cannot enter the nucleus to initiate the transcription of related genes. Second, ARE is a key regulator of NRF2-mediated SLC7A11 activation, and the ARE-NRF2 interaction is a p53-independent enhanced ferroptosis sensitivity approach.

A previous study indicated that NRF2 is an important and novel transcriptional regulator of ferroptosis in hepatocellular carcinoma (HCC) cells, and NRF2 activation can inhibit ferroptosis in HCC cells [67–69]. First, p62-mediated degradation of KEAP1 contributes to the activation of NRF2 during ferroptosis. Second, the genes quinone oxidoreductase 1 (NQO1), heme oxygenase 1 (HO-1), and FTH1 regulated by NRF2 inhibit ferroptosis by inhibiting iron metabolism and lipid peroxidation [67]. Inhibition of the p62-KEAP1-NRF2 antioxidant signaling pathway can significantly enhance the anticancer activity of erastin and sorafenib in liver cancer cells in vivo and in vitro [67]. The detailed functional characterization of this pathway may provide references for the treatment of liver cancer. The findings of Chen et al. showed that ARF inhibits the ability of NRF2 to transcriptionally activate its target gene *SLC7A11* in vivo and in vitro and regulates the ferroptosis response in a p53-dependent and p53-independent manner. The ARF–NRF2 interaction is essential for ARF to inhibit p53-dependent tumor growth [70, 71]. Therefore, these results reveal a new mechanistic role of ARF–NRF2 interaction in controlling the growth of cancer cells in vivo (Fig. 3).

Finally, gene inactivation of tumor suppressor NRF2 makes cancer cells in orthotopic malignant mesothelioma mouse models more sensitive to ferroptosis. The results show the role of cell–cell interaction and intracellular NRF2-YAP signaling pathway in determining ferroptosis and show that malignant mutations in the NRF2-YAP signaling pathway can predict the response of cancer cells to future induction therapy for ferroptosis [72].

### p53

Recent studies suggest that p53 plays an important role in controlling metabolism and ferroptosis and that p53 in normal cells can negatively regulate lipid synthesis and glycolysis and positively regulates oxidative phosphorylation and lipid catabolism. In tumor cells, mutant p53 positively regulates lipid synthesis and glycolysis. Therefore, in normal tissues, p53 tends to positively regulate ferroptosis, whereas mutant p53 makes tumor cells more sensitive to ferroptosis.

**Fig. 4:**
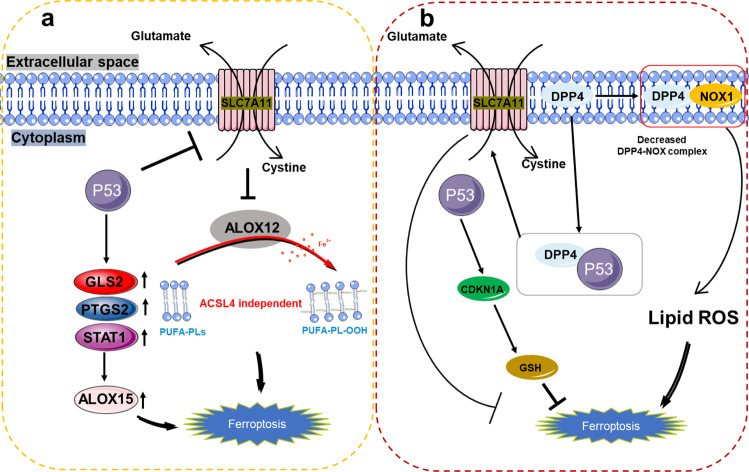
The various roles of p53 in ferroptosis. **a** P53 can further promote the upregulation of ALOX15 by promoting the expression of GLS2, PTGS2, and STAT1, and ultimately promote ferroptosis, or p53 can indirectly activate the function of ALOX12 by inhibiting the transcription of SLC7A11, leading to ALOX12-dependent ferroptosis after ROS stress, this pathway mediated by ALOX12 and independent of ACSL4, promotes the peroxidation of polyunsaturated fatty acid-containing phospholipids (PUFA-PLS). **b** P53 can also inhibit the generation of cellular lipid-reactive oxygen species by competitively binding to DPP4 with NOX1; at the same time, the P53-DPP4 complex promotes the expression of SLC7A11 and CDKN1A to inhibit ferroptosis.

p53 plays a dual role in regulating ferroptosis. On the one hand, it promotes ferroptosis by inhibiting the expression of SLC7A11 or promoting the expression of metabolism-related genes spermidine/Spermine N1-acetyltransferase 1 (*SAT1*) and glutaminase 2 (*GLS2*) [73–76]. On the other hand, p53 inhibits ferroptosis by inhibiting the activity of dipeptidyl peptidase 4 (DPP4) or inducing the expression of cyclin-dependent kinase inhibitor 1A (CDKN1A/p21) [77], both indicate the importance of p53 as a regulator of metabolism-related genes in ferroptosis (Fig. 4).

However, p53 activation has no evident effect on the function of GPX4, which shows that it does not induce ferroptosis through GPX4 [73]. In addition to the regulation of GPX4, the level of lipid peroxides in cells can also be regulated by lipoxygenase [17, 78]. Knockout of *ALOX12* by CRISPR/Cas9 can also inhibit cell ferroptosis. These studies indicate that ALOX12 is necessary for p53-mediated ferroptosis [74].

### NUPR1

The stress response gene, nuclear protein 1, transcriptional regulator (NUPR1) is a multifunctional stress-inducing protein, which is produced under a variety of environmental pressures, including oxidative damage and unfolded protein response. Oxidative stress activates NUPR1 by both ER stress (METH [79] and H2O2 [80]) and non-ER stress pathways, NUPR1 regulates ferroptosis via iron metabolism, ROS homeostasis, and the GSH/GPX4 pathway [81, 82]. An increase in intracellular ROS is a major signal that reacts with intracellular iron to produce more active ROS-type hydroxyl radicals (HO•) that trigger ferroptosis [81]. The importance of NUPR1 in maintaining mitochondrial function is now clear. Mitochondrial membranes, like cell membranes, are rich in polyunsaturated fatty acids (PUFAs) and are also susceptible to high levels of ROS [83]. NUPR1 inactivation can regulate mitochondria-related ferroptosis through mitochondrial dysfunction, decreased antioxidant capacity, and increased accumulation of endogenous iron content [84].

As a stress-inducing protein, NUPR1 is overexpressed in various malignant tumors. Sorafenib, an ferroptosis inducer, can induce ferroptosis by inhibiting System Xc-, also increases the expression level of NUPR1 [85]. In addition, other studies have found that various iron inducers, such as Erastin, and RSL3, strongly activate NUPR1, supporting its protective effect on iron [86]. Recently, NUPR1 can transmediate the gene encoding lipocalin 2 (LCN2) to reduce iron-induced oxidative damage and inhibit ferroptosis. Blocking NUPR1-dependent LCN2 expression significantly increased intracellular iron concentration and subsequent oxidative damage, including lipid peroxidation and DNA damage [87]. In general, NUPR1 is critical in regulating mitochondrial-related ferroptosis, either genetic or pharmacological inhibition of NUPR1 presents tumor-killing activity.

### Hippo signaling pathway

The Hippo signaling pathway regulator transcription regulator 1 (TAZ) regulates ferroptosis through epithelial membrane protein 1 (EMP1)-NOX4, which means that ferroptosis may be a therapeutic target for renal cell carcinoma and other TAZ-activated tumors [88]. TAZ affects the levels of EMP1 and NOX4 and causes lipid peroxidation and ferroptosis. Therefore, TAZ activation can enhance the sensitivity of ferroptosis and promote ferroptosis. In addition, YAP/TAZ is also regulated by metabolic pathways, which may help explain the important role of glutamine metabolism in ferroptosis [89]. In 1935, Hans Krebs proposed the famous tricarboxylic acid cycle (TCA), pointing out the importance of glutamine metabolism in animals [90]. Tumors can control ROS levels through products produced by the glutamine metabolic pathway, and the most important pathway for glutamine to control ROS is glutathione synthesis [91]. Glutamine is transported to cells through the transporter SLC1A5 (member of solute carrier family 1 neutral amino acid transporter 5), and glutamate is generated under the catalysis of glutaminase (GLS or GLS2) as one of the raw materials for glutathione synthesis [92]. Glutamic acid can be catalyzed by glutamase (GLUD) or transaminase to produce α-ketoglutaric acid, and eventually to acetyl-CoA, which can be used for the direct synthesis of lipids [93]. In addition, TAZ-EMP1-NOX4 and ZEB1-GPX4 may represent two different ways to connect chemotherapy resistance and ferroptosis, and they may play a role in providing resistance in different environments [88].

In epithelial cells, E-cadherin inhibits ferroptosis by mediating the interaction of intracellular NF2 (also called merlin) and Hippo signaling pathway while antagonizing the signal axis mediated by E-cadherin promotes ferroptosis through YAP [72]. The cadherin–Merlin–Hippo–YAP signaling axis often undergoes mutations in cancer. When this signaling pathway is antagonized, the protooncogene transcription coactivator YAP will be upregulated, leading to an increase in the key ferroptosis factors ACSL4 and TFRC, thereby inducing ferroptosis in cancer cells [88].

### Epithelial–mesenchymal transition

Both epithelial–mesenchymal transition (EMT) and ferroptosis can be regulated by epigenetics. In tumor cells, the epigenetic reprogramming of EMT makes head and neck cancer (HNC) cells more sensitive to ferroptosis [94]. To obtain more mesenchymal characteristics [95], cells use SIRT1 induction or miR-200 family inhibition, causing metastasis in cancer cells with interstitial properties [96, 97]. Viswanathan et al. [12] reported that in some cancer cells drug resistance occurs mainly because cancer cells are in the “quasi-stable state” with mesenchymal characteristics. In this state, the GPX4 is active in cells, and lipid peroxides are reduced, therefore leading to ferroptosis resistance. After knocking out the *ZEB1* gene related to lipid uptake, aggregation, and migration, the sensitivity to ferroptosis and susceptibility to ferroptosis inducers was significantly increased, leading to drug-resistant cancer cell death [78, 98]. Therefore, decreased E-cadherin expression or increased *ZEB1* expression leads to increased sensitivity to ferroptosis inducers [72, 99]. EMT regulation of cancer cells is a promising therapeutic strategy to promote the anticancer effect of ferroptosis inducers.

## Noncoding RNA and tumor cell ferroptosis

Noncoding RNAs (ncRNAs) can be divided into micro RNAs (miRNA), long noncoding RNAs (lncRNAs), and circular RNAs (circRNA) [4, 100, 101]. They are widely involved in the regulation of gene expression in cells [102–104], and play an important role in the occurrence and development of cancer [105, 106]. At present, the most widely studied ncRNAs related to ferroptosis are mainly miRNA, followed by lncRNAs and circRNAs. ncRNA can directly target ferroptosis-related molecules or downstream target genes and proteins in the form of miRNA–mRNA, IncRNA–miRNA, IncRNA–mRNA, circRNA–miRNA, and other regulatory networks to affect ferroptosis in tumor cells. Existing studies have partially revealed the mechanism of ncRNA regulating ferroptosis and other cell death in the context of tumor, and the intricate regulatory network of ncRNA may become the “hub” of various signaling pathways related to different cell death modes.

### miRNA and ferroptosis

miRNAs may regulate ferroptosis by affecting the expression of ROS [107]. For example, miR-206 induces ROS accumulation by binding to superoxide dismutase 1 (SOD1) mRNA [108], miR-155 increases ROS production by inducing FOXO3a deficiency [109]. While miR-25 and miR-448-3p reduce ROS levels by targeting nicotinamide adenine dinucleotide phosphate oxidase (NOX) [110, 111], tumor-associated fibroblasts (cancer-associated fibroblasts, CAFs) inhibit the ferroptosis in cancer cells by secreting exosomal miR-522 to target ALOX15 and blocking the accumulation of lipid ROS [112]. miR-9 regulates ferroptosis by targeting glutamate oxaloacetate transaminase 1 (GOT1), then reduce ROS levels in melanoma cells [113]. Polyunsaturated fatty acids (PUFAs), miR-3595 [114], miR-205 [115], and miR-224-5p [116] can reduce the expression of ACSL4. miR-150-5p [117] targets the downregulation of SLC38A1 to inhibit ferroptosis, whereas some miRNAs may promote ferroptosis by targeting SLC7A11. For example, miR-375, miR-27a, and miR-26b can inhibit the transcription of SLC7A11 mRNA [118–120]; miR-182–5p and miR-378a-3p negatively regulate the expression of GPX4 and SLC7A11 by directly binding the 3' UTR of GPX4 and SLC7A11 mRNA [121]; and miR-137 directly targets glutamine transport SLC1A5 to regulate ferroptosis negatively in melanoma cells [122].

Some miRNAs can induce ferroptosis by regulating the expression of NRF2. First, miR-7 and miR-200A can easily induce the activation of the NRF2 pathway by inhibiting the expression of Keap1 [123, 124]. Second, miR-101 and miR-455 can be targeted by Cullin-3 (CUL3) [125, 126] to promote the nuclear accumulation of Nrf2. According to existing research results, miRNAs are also involved in iron output, storage, utilization, and absorption. miR-20a and miR-485–3p can reduce iron output by targeting *FPN* genes [127, 128], miR-200b, and miR-let-7d effectively reducing iron accumulation by inhibiting the expression of FTH, DMT1, and iron response element (IRE) [129, 130]. miR-335 increases iron release, lipid peroxidation, and ROS accumulation by degrading FTH1, and reduces mitochondrial membrane potential (MMP), thereby promoting ferroptosis [131] (Table 1).

**Table 1:** miRNA associated with ferroptosis.

miRNA	Target	Mechanisms of action	Function	Reference
miR-206	SOD1	Significantly induced ROS accumulation	Promote	[108]
miR-155	Foxo3a	Induced ROS accumulation	Promote	[109]
miR-25, miR-448–3p	NOX	Decreases ROS level	Suppress	[110, 111]
miR-522	ALOX15	Block the lipid ROS accumulation	Suppress	[112]
MiR-3595	ACSL4	Targeting ACSL4 3’UTR, decreases its level	Suppress	[114]
miR-205	ACSL4	Decreases ACSL4 level	Suppress	[115]
miR-224–5p	ACSL4	Decreases ACSL4 level	Suppress	[116]
miR-150–5p	SLC38A1	Decreases SLC38A1 level	Suppress	[117]
miR-375, miR-27a, miR-26b	SLC7A11	Inhibit transcription of SLC7A11	Promote	[118–120]
miR-182–5p, miR-378a-3p	GPX4, SLC7A11	Targeting GPX4, SLC7A11 mRNA 3’UTR, decreases their level	Promote	[121]
miR-137	SLC1A5	Inhibiting glutaminolysis and malondialdehyde accumulation	Suppress	[122]
miR-7, miR-200a	Keap1	Induce activation of the Nrf2 pathway	Suppress	[123, 124]
miR-101, miR-455	Cullin-3	promote Nrf2 nuclear accumulation	—	[125, 126]
miR-20a, miR-485–3p	FPN	Reduce iron output	Suppress	[127, 128]
miR-200b, miR-let-7d	FTH, DMT1	Inhibit IRE expression, reduces iron accumulation	—	[129, 130]
miR-9	GOT1	miR-9 suppressed GOT1, reduced erastin- and RSL3-induced ferroptosis	Suppress	[113]
miR − 335	FTH1	Enhanced ferroptosis through the degradation of FTH1	Promote	[131]

### LncRNA and ferroptosis

ELAVL1 (ELAV-like RNA-binding protein 1) is highly expressed in many human tumors and regulates eukaryotic gene expression at the post-transcriptional level, which can enhance RNA stability [132, 133]. Wang et al. found that LINC00336 is an oncogene that combines with ELAVL1 to promote tumor cell proliferation, inhibit ferroptosis, and induce tumor formation in an ELAVL1-dependent manner [134]; LINC00618 interacts with lymphoid-specific helicase (LSH) to reduce the expression of SLC7A11, thereby inhibiting ferroptosis [135]. Erastin upregulated the expression of lncRNA GABPB1-AS1, inhibited the translation of GABPB1 and the expression of the peroxidase gene, leading to the accumulation of ROS and MDA, and promoting tumor cell death, suggesting that GABPB1-AS1 may be the key to Erastin-induced ferroptosis [136, 137] (Table 2).

**Table 2:** lncRNA associated with ferroptosis.

circRNA	Target	Mechanisms of action	Function	Reference
LINC00336	ELAVL1	Inhibiting ferroptosis in an ELAVL1-dependent manner	Suppress	[134]
LINC00618	LSH	Decreases SLC7A11 level	Suppress	[135]
Lnc GABPB1-AS1	GABPB1	Induced ROS and MDA accumulation	Promote	[136]

### CircRNA and ferroptosis

CircRNAs participate in the ferroptosis process of tumor cells through the competitive endogenous RNA (ceRNA) pathway. For example, CircABCB10 inhibits ferroptosis and apoptosis of rectal cancer cells by regulating the miR-326/CCL5 axis, providing a potential therapeutic target for the treatment of rectal cancer [138]. CircIL4R acts as a miR-541–3p sponge to regulate its target gene *Gpx4*; thus, circIL4R acts as a tumor promoter and ferroptosis inhibitor in liver cancer through the miR-541–3p/Gpx4 network [139]. Circ-TTBK2 uses sponge miR-761 to target ITGB8 to regulate the proliferation, invasion, and ferroptosis of glioma cells, providing a promising biomarker for the clinical treatment of human glioma [140]. The circular RNA clARS regulates the ferroptosis of HCC through the binding protein ALKBH5 [141]. To date, there have been only a few studies on circRNA in ferroptosis, which is an emerging field that needs to be further explored (Table 3).

**Table 3:** circRNA associated with ferroptosis.

circRNA	Target	Mechanisms of action	Function	Reference
CircABCB10	miR-326/CCL5	CircABCB10 silence promoted ferroptosis by regulating the miR-326/CCL5 axis	Promote	[138]
CircIL4R	miR-541–3p/Gpx4	circIL4R served for ferroptosis inhibitor by the miR-541–3p/GPX4 network	Suppress	[139]
Circ-TTBK2	miR-761/ITGB8	Circ-TTBK2 regulated cell ferroptosis via targeting ITGB8 by sponging miR-761	Suppress	[140]
Circ clARS	ALKBH5	Circ cIARS positively regulating sorafenib-induced ferroptosis through suppressing the ALKBH5-mediated autophagy inhibition	Promote	[141]

In recent years, researchers have detected some ncRNAs associated with ferroptosis in tumor cells. However, the specific mechanism has not been discussed yet, and there are still many obstacles in the clinical treatment of ferroptosis dependent on ncRNAs. The information summarized in our paper is not enough to support the application of ferroptosis inducers in cancer, more ncRNAs identification and further studies are needed. Downstream molecules regulated by ncRNAs, P53, HSPB1, NRF2, and NOX2, key regulators of ferroptosis, are also involved in the regulation of other cell death types [65], suggesting that ferroptosis and other types of cell death are independent and related. Further exploration of the interregulation of various types of cell death can provide insights into the role of regulatory cell death in cancer. Although it has been reported in the artical, ncRNAs may become markers to filter cancer patients who are fit for ferroptosis therapy and become therapeutic targets of ferroptosis inducers [142], there is no mature technique for ncRNA-mediated tumor therapy. To further understand the factors influencing the direction of ferroptosis regulated by these molecules, fully exert the upstream regulatory role of ncRNA in the signaling pathway, promote the pathway of ncRNA promoting ferroptosis in tumor cells, and inhibit its negative regulation of ferroptosis.

## Ferroptosis in tumor therapy

Targeted cell death is a common approach in tumor therapy [143]. Since ferroptosis inducers have the possibility of targeting cancer cells specifically, the use of ferroptosis-inducing drugs can improve the antitumor efficacy of these drugs [27]. In the course of chemotherapy or targeted therapy for cancer patients, drug resistance is a key problem to be solved urgently. Viswanathan et al. found that a drug-resistant high mesenchymal state is dependent on a GPX4-regulated lipid peroxidase pathway that protects cells against ferroptosis [78]. Tumor cells may significantly enhance their oxidative stress defense ability by negatively regulating iron ions, thereby achieving drug-resistant survival. By inducing tumor cell ferroptosis, it may be possible to reverse chemotherapy or targeted therapy drug resistance [144–146]. At present, ferroptosis inducers used in clinical tumor treatment primarily inhibit the activity of System X_C_- and GPX4. In addition, some natural products play an important role in inducing ferroptosis in tumor cells (Table 4).

**Table 4:** Preclinical drugs associated with ferroptosis.

Preclinical drugs	Mechanisms of action	Clinical/preclinical findings	Reference
Erastin	Inhibit System Xc-, GSH depletion	Gastric cancer treatment	[112]
Sulfasalazine	Inhibit System Xc-, GSH depletion	Ovarian cancer and breast cancer treatment	[149, 150]
RSL3	GPX4 inactivation	Ovarian cancer	[150]
ML162	Inhibit GPX4, induces lipid peroxidation	Induced head and neck cancer cell death	[161]
ML210	Covalently modified selenocysteine of GPX4	Mesenchymal-high cancers	[163]
FIN56	Deplete CoQ_10_, decreases GPX4 level	Breast carcinoma cells	[166]
Buthionine sulfoximine (BSO)	Decreases GSH and GPX4 level	Pancreatic cancer	[170]
Dihydroartemisinin	Induces ROS, decreases GSH and GPX4 level	Glioma cells	[174]
Artemisinin	Induces iron-dependent death	Head and neck carcinoma cells	[175]
Artesunate	Induces lipid peroxidation	Hepatocellular carcinoma	[176]
Sorafenib	Inhibit System Xc-, GSH depletion	Hepatocellular carcinoma and lung adenocarcinoma	[155, 156]
FINO2	Induces lipid peroxidation	Renal cancer cells	[186]
Statins	Deplete CoQ_10_, decreases GPX4 level	Triple-negative breast cancer	[179]
BAY-87–2243	increased cellular ROS levels, stimulated lipid peroxidation, and reduced glutathione levels	Melanoma cells	[185]
lapatinib	Disrupt iron transport or inactivates GPX4	Breast cancer and NSCLC cells	[181, 182, 184]
Withaferin A	Targets KEAP1 or inactivates GPX4	Neuroblastoma and myeloma cells treatment	[171, 172]

## Ferroptosis-inducing drugs used for tumor treatment

### Inhibition of the System XC- activity

The main function of System X_C_- is to transfer glutamate from inside the cell to the outside and transfer the extracellular cystine into the cell, and the transferred cystine is used for the synthesis of glutathione in the cell. When System Xc- is inhibited by ferroptosis inducers, glutathione synthesis is reduced. Subsequently, GPX4 is unable to use glutathione to reduce lipid peroxides, causing cell ferroptosis (Table 5).

**Table 5:** The applications of nanomaterials in targeting tumor ferroptosis.

nanomaterials	Target	Mechanisms of action	Reference
SRF@FeIIITA	GPX4	Inhibit GPX4 enzyme for ferroptosis initiation	[201]
AMSNs	GSH, GPX4	Highly efficient glutathione (GSH) depletion ability	[202]
FeGd-HN@Pt@LF/RGD2	GPX4	Accelerate Fenton reaction and generates ROS to induce ferroptosis	[203]
SPFeN	GPX4	Generates hydroxyl radicals and accelerates the Fenton reaction	[204]
Fe_3_O_4_-PLGA-Ce6	GSH, GPX4, SLC7A11	Accelerate Fenton reaction and generates ROS to induce ferroptosis	[205]
LDL-DHA	GPX4	Experience pronounced lipid peroxidation, depletion of glutathione, and inactivation of GPX4	[210]

Erastin is the first identified compound used to induce ferroptosis. Erastin selectively acts on tumor cells carrying the oncogene RAS; after acting on the tumor cell surface System X_C_- [112], it directly binds to mitochondrial voltage-dependent anion channel 2 (VDAC2) and induces mitochondrial damage that produces ROS in an NADH-dependent manner to inhibit GSH synthesis. Erastin induces cell death through the RAS–RAF–MEK pathway in some tumor cells expressing ferroptosis activating mutations [147]. In addition, Erastin strongly enhances the effect of wild-type epidermal growth factor receptor cells by inducing ROS-mediated caspase-independent cell death [148].

Sulfasalazine (SAS) also acts on System X_C_- to inhibit the uptake of cystine [149], causing chronic depletion of glutathione in cells, thereby destroying the redox defense of cells, hindering tumor growth, and inducing tumors ferroptosis [149–153].

Sorafenib induces ferroptosis by targeting the cystine/glutamate anti-transport system Xc- [154–157], an effect that impinges on cystine uptake, thereby preventing subsequent synthesis of glutathione (GSH), the major intracellular antioxidant, preventing GPX4 activation [158, 159], and can trigger ER stress and ferroptosis.

### Inhibition of the GPX4 activity

There are some ferroptosis inducers that block the intracellular antioxidant enzyme GPX4 through endogenous pathways. For example, RSL3, an activator of ferroptosis, does not depend on VDAC2/3 and is selective for tumor cells carrying tumorigenic RAS. Based on affinity chemical proteomics, the chloroacetamide part of the RSL3 structure is essential for its activity. The alkylation of selenocysteine directly inactivates GPX4 and induces lipid peroxidation, thereby inducing ROS production [23, 150, 160]. The other ferroptosis inducer, ML162 [161] and ML210, has the same effect as RSL3 in inducing ferroptosis and inhibiting the activity of GPX4. The ML210 small molecule is likely to exert its inhibitory effect by covalently modifying the selenocysteine of GPX4 [162, 163].

FIN56 was discovered through the modulation map of 56 caspase-independent lethal compounds. Idebenone is the only inhibitor of ferroptosis caused by FIN56 [164]. The oxime group in the structure of FIN56 is essential for the induction of ferroptosis, and the hydrophobicity of the piperidine group affects the potential of ferroptosis. There are two different ways in which FIN56 can induce ferroptosis. First, it uses the activity of acetyl-coenzyme A carboxylase (ACC) to degrade GPX4; second, FIN56 binds and activates squalene synthase (SQS). SQS is an enzyme involved in the synthesis of cholesterol, which leads to the depletion of the endogenous antioxidant Coenzyme Q10 (CoQ10), a non-steroidal metabolite in the glutaric acid pathway. This process enhances the sensitivity of ferroptosis induced by FIN56 [159, 164–166].

Glutathione is a tripeptide composed of three amino acids (cysteine, glutamic acid, and glycine). Buthionine sulfoximine (BSO) can selectively inhibit r-glutamylcysteine synthase (R-GCS), thus preventing the synthesis of dipeptides from glutamic acid and cysteine. Therefore, as a rate-limiting enzyme in the synthesis of glutathione (GSH), BSO can inhibit the synthesis of reduced glutathione, reduce the activity of GPX4, and promote ferroptosis [167–170].

Withaferin A, as a steroid isolated from *Withania somnifera*, is a natural ferritin inducer for neuroblastoma, dose-dependently either activates the NRF2 pathway through targeting of Kelch-like ECH-associated protein 1 (KEAP1) or aspartate aminotransferase (GPX4) to induce ferroptosis [171, 172].

### Other drugs induce ferroptosis

Artemisinin is a natural product of sesquiterpenes and is an effective component of the dried stems and leaves of *Artemisia annua* in the *Compositae* family. Studies have found that artemisinin can exert anticancer effects by inducing iron-dependent death of tumor cells [173]. Mechanistic studies have shown that artemisinin can bind to transferrin to enhance its selectivity and cytotoxicity in cancer cells. In addition, artesunate, a derivative of artemisinin, has significant antitumor activity. Artesunate can increase iron concentration by increasing ferritin hydrolysis and inducing ferroptosis through iron dependence [174]. Under the induction of artesunate, the level of lipid peroxidation in hepatocellular carcinoma (SMMC-7721) cells increases, and the use of iron-chelating agent deferoxamine can eliminate the accumulation of intracellular lipids caused by artesunate peroxidation [175, 176]. After the intervention of ovarian cancer cells, the intracellular ROS level increased and the ferroptosis inhibitor (Fer-1) can significantly inhibit artesunate-induced cell death [177].

Statins stand out as promising candidates for the therapeutic induction of ferroptosis in chemoresistant cancer cells [178]. By reducing isopentenic pyrophosphate production in the mevalonate pathway, statins inhibit biosynthesis of selenoproteins such as GPX4 and CoQ10, thereby promoting ferroptosis or selectively inducing regulatory cell death in mesenchymal cells [71]. satins could effectively kill triple-negative breast cancer (TNBC) through induce ferroptosis [179].

Lapatinib is approved for the treatment of ErbB2-positive breast cancer and other cancers that overexpress ErbB2. In particular, it is used as a treatment for patients with advanced or metastatic ErbB2-positive breast cancer [180, 181]. Combining siramesine and lapatinib causes ferroptosis through iron transport disruption leading to increased ROS in breast cancer [182, 183], or targeting GPX4 might be a potential strategy to enhance antitumor effects of lapatinib in NSCLC cells [184].

BAY-87–2243 inhibits complex I (CI) of the mitochondrial respiratory chain then triggers ferroptosis of BRAFV600E melanoma cell lines [185]. On the other hand, In contrast to previously described iron inducers, FINO2 neither inhibits systemic XC -, nor directly targets the reductase GPX4 as erastin and RSL3 do, nor consumes the GPX4 protein as FIN56 does. In contrast, FINO2 both indirectly inhibits the enzyme function of GPX4 and directly oxidizes iron, ultimately leading to extensive lipid peroxidation [186, 187].

In addition to the preclinical drugs associated with ferroptosis mentioned above, some ferroptosis inhibitors are widely used in tumor research. Such as ferrostatin-1 and liproxstatin-1, both inhibitors work by inhibiting lipid peroxides, thereby inhibiting ferroptosis in tumor cells [188, 189]. Vitamin E, a fat-soluble Vitamin, is one of the main antioxidants and also inhibits ferroptosis by inhibiting the production of lipid peroxides [190]. Deferoxamine (DFO) [187], Ciclopirox [191], and Deferiprone [192] inhibit ferroptosis by deplete iron.

We summarize the mechanisms of ferroptosis in reversing drug resistance in preclinical studies. But there is still a long way to go before it can actually be used in patients. At the same time, we also face many challenges: the development of novel ferroptosis-inducing drugs requires consideration of drug toxicity and prevention of off-target effects to avoid other adverse reactions in patients; ferroptosis may be associated with a variety of pathological conditions—including acute kidney injury, tissue deficiency Blood and reperfusion injury and neurodegeneration, etc., in the process of inducing ferroptosis of tumor cells, it is also necessary to avoid other systemic adverse reactions in patients; In addition, since various types of cancer have different sensitivities to ferroptosis, we are temporarily unable to confirm whether the therapeutic strategy of inducing tumor cells ferroptosis in patients is universal? And which drugs are most suitable for clinical treatment? We also need to identify the target patient population most likely to benefit from this strategy.

## Nanomaterials used for the treatment of tumor ferroptosis

In recent years, researchers have tried to combine bio-nanotechnology with ferroptosis to develop candidates with a stronger antitumor effect [193, 194]. The delivery of nano-drugs is based on engineering technology. Nanoparticles are used to deliver and control drug release and adjust the intracellular chemical reaction to affect the ROS levels, thereby improving the pharmacokinetic properties of the drug [58, 195, 196]. Nanomaterials can be used to supplement exogenous lipids in tumor cells to increase the accumulation of intracellular lipid peroxides, promote ferroptosis, and achieve the goal of curing cancer [197]. The current emerging nanotherapies mainly focus on inhibiting the expression of GPX4 in tumor cells, increasing the accumulation of ferrous/iron ions in tumor cells, and regulating lipid peroxidation [198–200]. This process primarily involves triggering or promoting the Fenton response in tumor cells [198].

### Inhibition of GPX4 expression

Currently, nanomaterials can be used to disrupt pathways related to the activity of GPX4 to induce ferroptosis and drive cancer therapy [201, 202]. Some nanomaterials, such as sorafenib, are encapsulated into network-like nanostructures composed of Fe^3+^ and tannic acid (TA) [201]. Sorafenib is a typical small-molecule System Xc- inhibitor. It inhibits GPX4, leading to tumor-specific ferroptosis, and TA is used to chemically reduce Fe^3+^ to Fe^2+^ and continuously supply Fe^2+^ to maintain the iron redox cycle and maintain the Fenton reaction [201]. Shen et al. by using lactoferrin (LF) and RGD dimer (RGD2)-coupled cisplatin (CDDP) Fe_3_O_4_/Gd_2_O_3_ hybrid nanoparticles FeGd-HN@Pt@LF/RGD2 successfully combined and delivered Fe^2+^, Fe^3+^, and H_2_O_2_ (the reactants involved in the Fenton reaction) to the tumor sites. Their local concentration was increased to accelerate the Fenton reaction, significantly improving the efficacy of in situ brain tumor ferroptosis treatment [203]; conversely, targeting GPX4 by nanodrug delivery systems (nano-DDS) of small-molecule inhibitors can overcome the shortcomings of rapid systemic clearance and poor tumor targeting. Similar to the classic ferroptosis inducer Erastin, nano-DDS can inactivate GPX4 by depleting the intracellular GSH substrate. Shuaifei Wang et al. synthesized arginine-rich manganese silicate nanobubbles (AMSNs) to target tumor cells by inducing ferroptosis [202].

### Increasing the accumulation of ferrous/iron ions in tumor cells

Iron-based nanomaterials can induce cell ferroptosis and provide an innovative method for cancer treatment. Current “ferroptosis” therapeutic nano preparations are often combined with other treatment methods, resulting in complex nanostructures and multi-metal compositions. Shasha He and others from Nanyang Technological University in Singapore reported the progress on the development of iron-chelated semiconductor multi-composite nanoparticles (SPFeN) under the guidance of photoacoustic (PA) imaging for the treatment of cancer using photothermal “ferroptosis” [204]. Professor Song Yang and Associate Professor Zhu Xiaokang from Southwest University designed a poly-nanosystem Fe_3_O_4_-PLGA-Ce6 coated with PLGA, containing iron oxide (Fe_3_O_4_) and photosensitizer Ce6, and used it to synergize ferroptosis–photodynamics anticancer treatment. Fe_3_O_4_-PLGA-Ce6 nanosystem can dissociate in acidic TME and release ferrous/iron ions and Ce6. Subsequently, the released ferrous/iron ions will react with excess hydrogen peroxide in the cell to produce a Fenton-like reaction generating hydroxyl free radicals (•OH), and induce ferroptosis of tumor cells [205]. In addition, in the xenograft model, ultra-small silica nanoparticles were shown to induce ferroptosis by increasing the transport and accumulation of iron in cells and inhibit tumor growth [206, 207].

### Exogenous regulation of lipid peroxidation in tumor cells

Ferroptosis is closely related to the accumulation of lipid hydrogen peroxide in cells, which is mainly derived from PUFAs of membrane phospholipids under oxidative stimulation [144, 208]. Therefore, the use of nano-DDS to exogenously supplement cancer cells with extra polyunsaturated fatty acids to exogenously regulate lipid peroxidation is also an effective strategy to improve the therapeutic effect of ferroptosis-driven cancer treatment [209–211]. Zijian Zhou et al. found that in triple-negative breast cancer (TNBC) cells, there is a close relationship between the level of PUFAs in the cell and ferroptosis [211]. Among them, conjugated linolenic acid showed the strongest activity as a ferroptosis inducer. These results provide evidence that PUFAs exert antitumor activity by inducing ferroptosis.

Combining ferroptosis-inducing inducers with bio-nanotechnology for tumor therapy has broad application prospects [212], but there are still many problems to be solved in clinical ferroptosis-based nanotherapy. First of all, the potential toxic and side effects of nanomaterials should be fully studied to ensure the safety of their application in clinical treatment; in addition, there is an urgent need to develop more precise targeted nanomaterials to ensure tumor-specific triggering of ferroptosis while avoiding off-target toxicity to normal tissues; as nanomaterial-mediated ferroptosis may vary greatly between animals and patients, a more complete assessment system is needed in clinical studies.

## Conclusions and perspectives

So far, researchers have elucidated some regulatory mechanisms and signal transduction pathways of ferroptosis [213, 214]. Anticancer strategies based on ferroptosis have been widely recognized, and there has been an upsurge in the development of anticancer drugs worldwide. Several natural products have been found to have the potential to be used as ferroptosis anticancer drugs. Therefore, the rational use of these mechanisms in the biomedical field to regulate cell ferroptosis and specifically induce tumor cell ferroptosis during tumor treatment is a promising cancer treatment strategy [215]. Ferroptosis inducers such as Erastin and RSL3 can synergistically induce tumor growth inhibition with anti-PD-L1 antibodies both in vitro and in vivo, and participate in immunotherapies [71]. Wang et al. found that in vitro culture with low cystine and in vivo data show that ferroptosis is involved in T cell-mediated cancer immunity [216]. Which also provoked a review of the relationship between ferroptosis mechanisms and immune system activation [217].

In addition, nanoparticles carrying chemicals or biological materials will provide the possibility to improve the efficacy of existing ferroptosis inducers and to develop new inducers for the treatment of cancer. Some nanoparticles can sensitize effective ferroptosis, produce mild immunogenicity, and improve the response rate of non-inflammatory tumors in cancer immunotherapy [218]. The biomimetic magnetic nanoparticles Fe3O4-SAS@PLT-mediated ferroptosis and immunotherapy is expected to provide great potential in treating tumor metastasis [219]. Typical strategies mainly focus on developing high-performance nanocatalysts and increasing intracellular reactants (such as H_2_O_2_ and iron ions) based on the Fenton reaction of nano-DDS [198, 200]. Several nanocatalysts have been developed to initiate the local Fenton response in tumors for cancer treatment. However, the long-term effects of nanoparticles on human health still need to be carefully evaluated.

The antitumor effect of ionizing radiation may be enhanced by triggering ferroptosis and ferroptosis inducers with effective radiosensitizers [71, 220–224]. Due to the non-apoptotic nature of ferroptosis, ferroptosis-based cancer treatments are expected to bypass the shortcomings of traditional therapies mediated by apoptotic pathways [225]. Targeting pathways that can regulate tumor cell ferroptosis is an emerging antitumor strategy because malignant tumor cells usually rely on oncogenic and survival signals, making them particularly vulnerable to ferroptosis. A better understanding of the regulatory mechanism and signaling pathways of ferroptosis and finding ways to help detect and track ferroptosis biomarkers will be an active research area in the next few years [32, 71].

## Data Availability

All data generated or analyzed during this study are included in this published article.
